# IGFBP7 Concentration May Reflect Subclinical Myocardial Damage and Kidney Function in Patients with Stable Ischemic Heart Disease

**DOI:** 10.3390/biom12020274

**Published:** 2022-02-08

**Authors:** Anna Lisowska, Anna Szyszkowska, Małgorzata Knapp, Magda Łapińska, Marcin Kondraciuk, Inga Kamińska, Tomasz Hryszko, Katarzyna Ptaszyńska-Kopczyńska, Karol Kamiński

**Affiliations:** 1 Department of Cardiology, Medical University of Bialystok, 15-276 Bialystok, Poland; annaszyszkowska92@gmail.com (A.S.); malgo33@interia.pl (M.K.); katarzyna.ptaszynskak@gmail.com (K.P.-K.); fizklin@wp.pl (K.K.); 2 Department of Population Medicine and Lifestyle Diseases Prevention, Medical University of Bialystok, 15-276 Bialystok, Poland; magda.lapinska@umb.edu.pl (M.Ł.); marcin.kondraciuk@umb.edu.pl (M.K.); 3 Department of Integrated Dentistry, Medical University of Bialystok, 15-276 Bialystok, Poland; inga.kaminska@umb.edu.pl; 4 2nd Department of Nephrology, Medical University of Bialystok, 15-276 Bialystok, Poland; tomasz.hryszko@umb.edu.pl

**Keywords:** ischemic heart disease, heart failure, IGFBP-7, NTproBNP, troponin T

## Abstract

The objective of this study was to determine the associations between insulin-like growth-factor-binding protein 7(IGFBP7) concentrations and concentrations of troponin T(TnT), N-terminal pro-B-type natriuretic peptide(NT-proBNP) and the parameters of kidney function in patients with stable ischemic heart disease(IHD). The IHD group consisted of 88 patients, and the population group comprised 66 subjects without a history of IHD. IGFBP7, TnT and NTproBNP concentrations were measured. The IGFBP7 value was considerably higher in the IHD group (1.76 ± 1 ng/mL vs. 1.43 ± 0.44 ng/mL, respectively, *p* = 0.019). Additionally, IHD subjects had a significantly higher concentration of TnT and NTproBNP. In both groups there was a significant correlation between IGFBP7 and serum parameters of kidney function (creatinine concentration: population gr. r = 0.45, *p* < 0.001, IHD gr. r = 0.86, *p* < 0.0001; urea concentration: population gr. r = 0.51, *p* < 0.0001, IHD gr. r = 0.71, *p* < 0.00001). No correlation between IGFBP7 and microalbuminuria or the albumin to creatinine ratio in urine was found. Moreover, there was a significant correlation between IGFBP7 concentration and markers of heart injury/overload-TnT and NT-BNP(r = 0.76, *p* < 0.001 and r = 0.72, *p* < 0.001, respectively). Multivariate regression analysis in joint both revealed that the IGFBP7 concentration is independently associated with urea, creatinine and TnT concentrations (R^2^ for the model 0.76). IHD patients presented significantly higher IGFBP7 concentrations than the population group. Elevated IGFBP7 levels are associated predominantly with markers of kidney function and myocardial damage or overload.

## 1. Introduction

Ischemic heart disease (IHD), due to the declining prevalence of hypertension and valvular heart disease, is the most common cause of heart failure (HF) in developed countries [1,2]. According to epidemiological data, IHD globally affects around 126 million individuals (1655 per 100,000), which is approximately 1.72% of the world’s population, and its prevalence is rising [3]. IHD contributes to both HF with reduced ejection fraction (HFrEF) and HF with preserved ejection fraction (HFpEF) and it is related with a poor prognosis [1]. In order to diagnose, assess and monitor patients with HF and IHD, markers of heart injury are measured. The most common are troponin T (TnT) and NTproBNP. Their elevated levels are proven to be associated with worse clinical outcomes and further development of HF, even in patients with a preserved ejection fraction [4,5,6]. However, their evaluation in daily medical practice is sometimes not sufficient to make an accurate diagnosis, and there is need to find new markers that help resolve this issue. Recently, more attention has been drawn to the effects of insulin-like growth-factor-binding protein 7 (IGFBP7) as a marker of cellular senescence, insulin resistance and atherosclerosis. IGFBP7 is a 30-kDa modular secreted protein, which is also called mac25, tumor adhesion factor (TAF), the prostacyclin-stimulating factor (PSF) or angiomodulin. IGFBP7 is a part of the insulin-like growth factor system, which is involved in the growth, proliferation and differentiation of human cells [7,8]. According to an increasing body of evidence, the insulin-like growth factor system could play an important role in the highly complex pathogenesis of atherosclerosis. IGF-1 (insulin-like growth factor-1) has been proven to stimulate angiogenesis and have anti-inflammatory and anti-apoptotic actions [9]. Moreover, it has indirect effects on the cardiovascular system by increasing insulin sensitivity. In the PRIME study a link between low levels of circulating IGF-1 and a high prevalence of IHD in healthy subjects has been suggested [10]. In our research we focused on IGFBP7, which interacts with IGF-1 and with insulin. By binding with IGF-1, it neutralizes its activity. What is more, it may interfere with the biological response of insulin, and it has many IGF-independent actions. Its role in atherosclerosis development remains unclear and requires further studies. According to our previous research, IGFBP7 seems to be a good marker of IHD occurrence—its concentration was significantly higher in patients with coronary lesions than in healthy subjects. However, its levels do not reflect IHD advancement [11].

The aim of the study was to determine the associations between IGFBP7 concentrations and concentrations of troponin T, NT-proBNP and serum parameters of kidney function in patients with stable IHD.

## 2. Materials and Methods

### 2.1. The Study Population

The IHD group included 88 patients (19 females—22%) with established IHD approximately a year after myocardial infarction or percutaneous coronary intervention. The population group—which has no history of IHD (65 subjects, 24 females, 38.8%)—was chosen based on age and gender from a larger cohort representative of the local population. The study group comprised 88 patients aged 63.2 ± 7.8 recruited to one of the cardiology centers taking part in multinational registry EUROASPIRE V assessing the efficacy of secondary prevention measures in Europe [12]. The criteria for recruitment and exclusion have been described previously [13]. In short, all patients had been hospitalized 6–18 months prior to their enrolment in the study. After enrolment, detailed medical history was taken as well as a large number of imaging and functional tests of cardiovascular system and carbohydrate metabolism. The control group (n = 65) was recruited from participants of the population-based cohort study Bialystok PLUS [14], which represents general local population in age, 20–80. We excluded patients with a history of cardiovascular disease (myocardial infarction, coronary or peripheral vascular intervention, stroke and peripheral vascular disease) and chose patients based on their age, gender and BMI to match the study group.

### 2.2. Biochemical Evaluation

In order to obtain analyzed biochemical parameters and IGFBP 7 concentration, the blood was drawn via a closed system of the Monovette type (Sarstedt Company, Nümbrecht, Germany). The biochemical parameters were determined within 2 h after the material’s collection. Troponin T (TnT) and NT-proBNP analysis was performed using electrochemiluminescence method on the Cobas e411 (ROCHE Diagnostics International Ltd., Rotkreuz, Switzerland). The blood samples (5 mL) used for IFGBP7 determination were left for 2 h at room temperature to allow clot formation. Then, they were centrifuged at 1000× *g* for 20 min at room temperature, and obtained supernatant serum was frozen and stored at −80 °C. The concentration of IGFBP7 was established with commercially available ELISA kit 7 (insulin growth-factor-binding protein; USCN Life Science Inc., Wuhan, Hubei, China). C-peptide was determined by electrochemiluminescence assay (ECLIA) on Cobas e411 equipment (ROCHE Diagnostics International Ltd., Rotkreuz, Switzerland).

In all participants oral glucose tolerance test (OGTT) and blood pressure and antropometric measurements were performed as described previously [13].

### 2.3. Statistical Analysis

The mean values and standard deviations for quantitative variables as well as the quantitative and percentage distribution for qualitative variables were calculated. Pearson’s correlation coefficient for categorical variables of normal distribution and Spearman’s correlation coefficient for variables not satisfying normal distribution criteria were calculated. To compare the groups, the statistical analysis of normal distribution variables estimated with the use of the Kolmogorov compatibility test was carried out using the unpaired Student’s test and the Mann–Whitney test for variables inconsistent with a normal distribution. The comparison of qualitative variables between the groups was performed using the Chi2 test. A multivariate analysis of the factors affecting IGFBP7 concentration was performed using general linear model A. Value of *p* < 0.05 was considered statistically significant. The statistical analysis was carried out using Statistica 12.0 PL software (StatSoft, Cracow, Poland).

## 3. Results

Patients in the IHD group presented a significantly higher concentration of IGFBP7 than those in the population group (1.76 ± 1 ng/mL vs. 1.43 ± 0.44 ng/mL, respectively, *p* = 0.019), as well as a significantly higher concentration of creatinine, urea, glucose, insulin, TnT and NTproBNP (detailed characteristics of the studied group are presented in Table 1).

**Figure 1:**
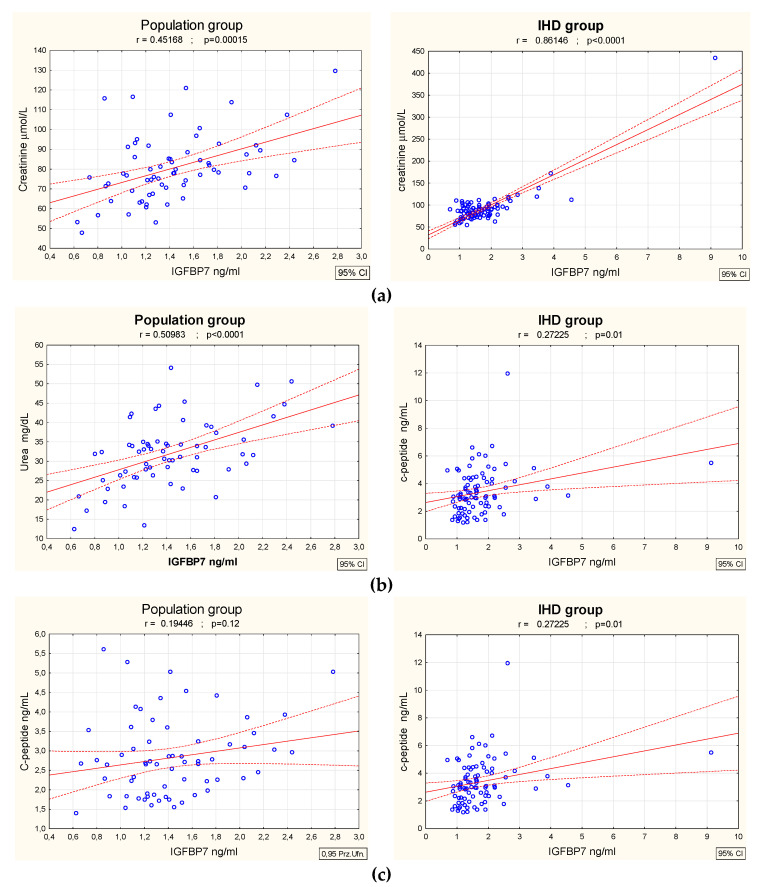
Correlations between concentration of IGFBP7 and concentrations of creatinine (**a**), urea (**b**) and c-peptide (**c**) in population group (n = 65) and ischemic heart disease patients (n = 88).

In both groups there was a significant correlation between IGFBP7 and serum parameters of kidney function (creatinine concentration: population gr. r = 0.45, *p* < 0.001, IHD gr. r = 0.86, *p* < 0.0001; urea concentration: population gr. r = 0.51, *p* < 0.0001, IHD gr. r = 0.71, *p* < 0.00001), as seen in Figure 1a,b. Interestingly, no correlation between IGFBP7 and microalbuminuria or the albumin to creatinine ratio in urine was found.

**Figure 2:**
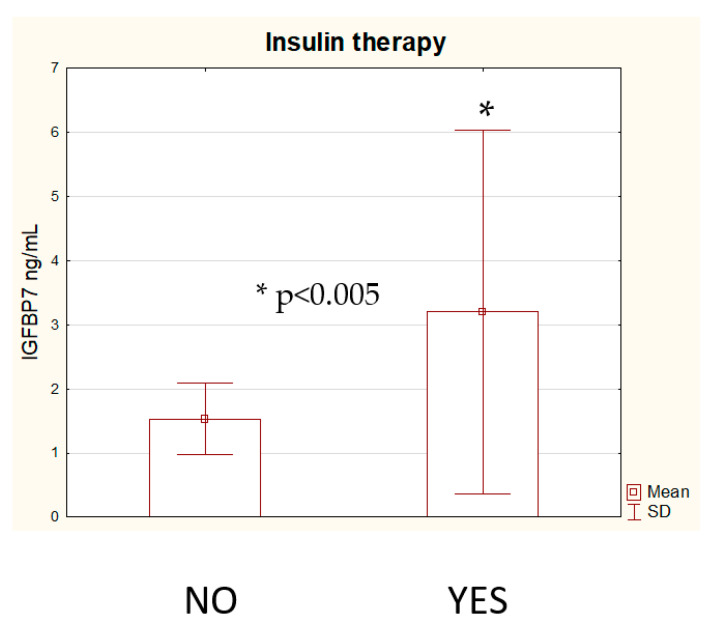
IGFBP7 concentration in patients on insulin therapy.

There was no significant correlation between IGFBP7 concentration and HBA1c levels in either group. Only the C-peptide concentration was considered statistically significant, but it was only weakly correlated with IGFBP-7 levels in patients with IHD (r = 0.27, *p* = 0.01, C-peptide r = 0.28, *p* < 0.001)—Figure 1c. Moreover, diabetic patients treated with insulin presented significantly higher IGFBP7 concentrations than diabetics treated with oral drugs (3.81 ± 3.2 ng/mL vs. 1.51 ± 0.5 ng/mL)—Figure 2.

**Figure 3:**
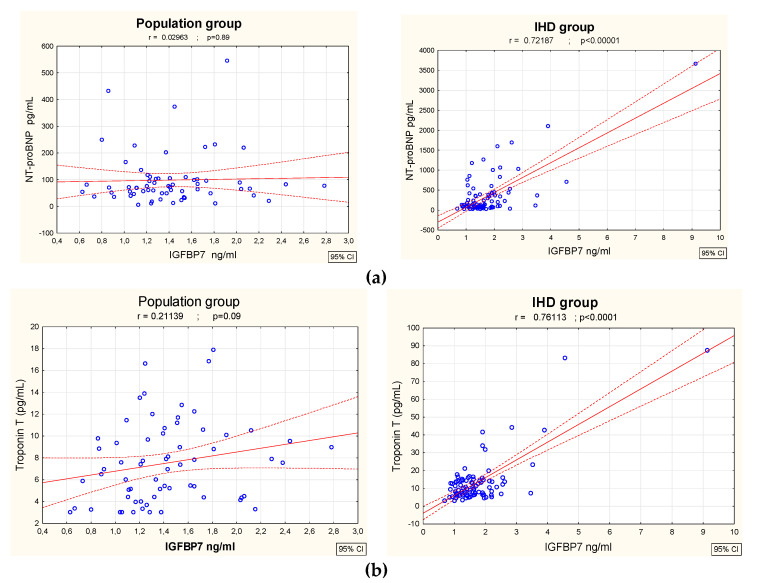
Correlations between concentration of IGFBP7 and concentrations of NTproBNP (**a**) and troponin T (**b**) in population group (n = 65) and ischemic heart disease patients (n = 88).

We also found that in the IHD group there was a significant correlation between IGFBP7 concentration and the markers of heart injury/overload, TnT and NT-BNP (r = 0.76, *p* < 0.001 and r = 0.72, *p* < 0.001; respectively), as seen in Figure 3a,b.

Multiple regression modeling of factors influencing IGFBP7 in the combined population of both groups showed that concentrations of urea, creatinine and TnT were independent predictors, while a diagnosis of ischemic heart disease and NT-BNP concentration did not have a statistically significant influence on the IGFBP7 concentration (Table 2). This multivariable model yielded very good prediction power (R^2^ = 0.77), and it decreased only slightly after the removal of variables that were not statistically significant (R^2^ = 0.76). Neither a previous diabetes diagnosis nor laboratory parameters of glucose metabolism (glucose, insulin and C-peptide concentrations in an oral glucose tolerance test) were found to be independently associated with IGFBP7 concentration in the multivariate analysis.

## 4. Discussion

IGFBP7 has been recently identified as a novel biomarker for heart failure and cardiac hypertrophy. Intensive research according to its role in developing and progressing obesity, diabetes, acute kidney injury (AKI), cancers, chronic obstructive pulmonary disease (COPD) and other diseases has been conducted [15,16,17].

IGFBP7′s role in atherosclerosis development is yet to be determined. In our previously published research, which is, as far as we know, the first one handling that issue, we observed that IGFBP7 seems to be a good marker of IHD occurrence [11]. Its levels were significantly higher in the group of patients with myocardial infarction (MI) and coronary artery disease (CAD) than in the control group of healthy subjects. However, there were no statistically significant differences in IGFBP7 concentration between patients with stable IHD and MI [11]. The results obtained from our current study, indicating significantly higher levels of IGFBP7 in the group of patients with IHD compared to the population group, confirm the possible role of this protein in atherosclerosis development.

An increased concentration of IGFBP7 is also associated with insulin resistance (IR) and the risk of metabolic syndrome (MetS) [7,18]. This protein has a high affinity to insulin (500 times higher compared to other IGFBPs) and competes with the insulin receptor (InsR) for binding insulin, which substantially reduces the serum level of free insulin, blocks the binding of insulin to the InsR and diminishes the physiological response to insulin. As a result, it contributes to insulin resistance, diabetes and cardiovascular diseases development [7,18]. Patients with MetS and IR have a significantly higher serum concentration of IGFBP7 than healthy individuals [7]. In our research we have observed that diabetic patients treated with insulin presented significantly higher IGFBP7 concentrations, which is consistent with previous findings on IGFBP7′s role in IR development. Moreover, we found that IGFBP7 levels significantly correlated with C-peptide concentration in patients with IHD, which has not been reported in any previous research. C-peptide is a short 31-amino-acid polypeptide that connects insulin’s A-chain to its B-chain in the proinsulin molecule. Measurements of its concentration reflect the actual insulin production through the pancreas. Interestingly, in our study there was no significant correlation between IGFBP7 concentration and HBA1C levels in either group of patients.

In our research we also found a significant correlation between IGFBP7 and serum parameters of kidney function (creatinine and urea) in both groups of patients. It is consistent with recent research, which has proven that IGFBP7, together with the tissue inhibitor of metalloproteinases-2 (TIMP-2), is a novel marker of tubular damage, and it can be used for the early detection of acute-kidney-injury (AKI)-endangered patients. Urinary TIMP2*IGFBP and IGFBP7 were proven to be the most accurate biomarkers for the prediction and renal outcome in patients with AKI [19,20,21]. However, we have not reported any correlation between IGFBP7 and the albumin to creatinine ratio in urine.

Elevated levels of IGFBP7 were also found in patients with heart failure [22,23]. Moreover, a combination of IGBP7 and NTproBNP correlated with shorter event-free survival [24]. In our study there was a significant correlation between IGFBP7 and NTproBNP concentration in patients with IHD. According to previous findings, it seems that left ventricular filling pressure may be an important trigger for IGFBP7 expression and release because of its strong independent association with an increased left atrial volume index (LAVI) [25,26]. We have not assessed ejection fraction (EF) in our research, but our previous findings showed no statistically significant differences of IGFBP7 concentrations between low-EF (<50%) and high-EF (>50%) patients [11].

An increase in high-sensitivity troponin T (hs-TnT) occurs not only in acute heart injury (such as MI or pulmonary embolism) but also in patients with stable chronic HF. According to previous research, elevated hs-Tn in this group of patients is caused by chronic low-grade cardiac ischemia [27]. It is consistent with our data that patients with IHD had higher TnT concentrations, which also significantly correlated with IGFBP7 levels. It seems that IGFBP7 could be considered as a plasma biomarker of chronic, ongoing myocardium damage, especially in patients with IHD, when ischemic cardiomyocytes are replaced by fibrous tissue.

To summarize our research, it seems that the multi-marker approach, combining measuring well-established cardiac-specific markers such as NTproBNP and troponin T with “new” ones such as IGFBP7 or galectin-3 [28], will become a daily routine in our future medical practice.

## 5. Conclusions

Elevated IGFBP7 levels are associated not only with established risk factors of heart failure such as coronary atherosclerosis, kidney function and glucose metabolism disturbances, but also with a marker of myocardial damage or overload, TnT.

IGFBP7 is a new promising marker in cardiovascular medicine, and its role should be further investigated, not only in respect to vascular changes but also heart failure and left ventricular dysfunction.

## Figures and Tables

**Table 1:** Characteristics of analyzed groups.

Variables	IHD (n = 88)	Population Group (n = 65)	*p*
Age (years)	63.2 (7.8)	61.2 (8.2)	NS
Male gender (n, %)	69 (78%)	41 (61.2%)	NS
BMI	29.8 (5.5)	28.7 (4.1)	NS
IGFBP7 (ng/mL)	1.76 (1.04)	1.41 (0.45)	0.019
Creatinine (µmol/L)	92.2 (41.4)	80.5 (16.8)	<0.0001
Urea (mg/dL)	38 (15.2)	31.9 (8.4)	<0.0001
Glucose (mg/dL)	114.7 (36.7)	95.9 (14.2)	<0.0001
Insulin (µU/mL)	15.03 (9.5)	12.8 (6.4)	<0.0001
C-peptide (ng/mL)	3.38 (1.6)	2.83 (1)	NS
TnT (pg/mL)	13.57 (13.6)	7.5 (3.7)	<0.0001
NT-proBNP (pg/mL)	355.2 (538.7)	98.2 (97.9)	<0.0001

**Table 2:** Multiple regression model of factors influencing concentration of IGFBP7 in combined groups.

Multiple Regression Analysis R^2^ for the Multivariate Model 0.77	Beta	*p*
Urea	0.45	<0.0001
Creatinine	0.23	<0.0001
TnT	0.23	<0.001
NT-BNP	0.1	0.055
Ischemic heart disease	−0.02	0.5

## Data Availability

Data may be available on reasonable scientific request to the senior author.

## Abbreviations

AHF—acute heart failure; AKI—acute kidney injury; BMI—Body Mass Index; CAD—coronary artery disease; COPD—chronic obstructive pulmonary disease; EF—left ventricular ejection fraction; HF—heart failure; HFpEF—HF with preserved ejection fraction; HFrEF—HF with reduced ejection fraction; hs-Tn—high-sensitivity troponin; IGF—insulin-like growth factor; IGFBP7—insulin-like growth-factor-binding protein 7; IGFBPs—insulin-like growth-factor-binding proteins; IGF-R—IGF receptor; IHD—ischemic heart disease; Ins-R—insulin receptor; IR—insulin resistance; LAVI—left atrium volume index; LV—left ventricular; MetS—metabolic syndrome; MI—myocardial infarction; NGAL—neutrophil-gelatinase-associated lipocalin; NSTEMI—non-ST-elevation MI; NT-proBNP—N-terminal pro-B-type natriuretic peptide; OGTT—oral glucose tolerance test, PCI—percutaneous coronary intervention; PSF—prostacyclin-stimulating factor; sST2—soluble suppression of tumorigenicity-2; STEMI—ST elevation MI; TnT—troponin T; TAF—tumor adhesion factor; TGF-β—transforming growth factor β; TIMP-2—Tissue inhibitor of metalloproteinases-2.
